# First Clinical Application of Repeated Boron Neutron Capture Therapy for Olfactory Neuroblastoma: Achieving Local Control Despite Regional Progression

**DOI:** 10.7759/cureus.101324

**Published:** 2026-01-12

**Authors:** Hung-Ruei Liao, Ming-Ying Lan, Ko-Han Lin, Fong-In Chou, Yi-Wei Chen

**Affiliations:** 1 Radiation Oncology, Taipei Veterans General Hospital, Taipei, TWN; 2 Otolaryngology - Head and Neck Surgery, Taipei Veterans General Hospital, Taipei, TWN; 3 Nuclear Medicine, Taipei Veterans General Hospital, Taipei, TWN; 4 Nuclear Science and Technology Development Center, National Tsing Hua University, Taipei, TWN; 5 Heavy Particles and Radiation Oncology, Taipei Veterans General Hospital, Taipei, TWN

**Keywords:** boron neutron capture therapy (bnct), multiple re-irradiation, -olfactory neuroblastoma, recurrent tumor, salvage radiotherapy

## Abstract

To evaluate the feasibility and clinical outcomes of two-course boron neutron capture therapy (BNCT) as a salvage treatment for multiply recurrent olfactory neuroblastoma (ONB), and to discuss the implications of intratumoral heterogeneity on treatment planning. A 50-year-old male with recurrent ONB with extensive intracranial invasion underwent BNCT after exhausting surgical and conventional radiotherapy options. Boronophenylalanine (BPA) was administered at 500 mg/kg. The patient received two courses of neutron irradiation at the Tsing Hua Open-Pool Reactor (THOR). Dosimetry was guided by 18F-fluciclovine PET imaging (serving as a surrogate for BPA biodistribution) and calculated using the simulation environment for radiotherapy applications (SERA) treatment planning system. The first BNCT course (April 2024, Tumor Dmax 49.2 Gy-Eq) resulted in significant tumor shrinkage; however, PET imaging revealed residual metabolic activity in centrally necrotic regions. Consequently, a second BNCT course was administered (March 2025, Tumor Dmax 40.73 Gy-Eq). The patient achieved a near-complete response at the primary skull base site with no high-grade adverse effects. Although local control was sustained as of November 2025, a new cervical lymph node metastasis was identified. BNCT serves as a potent and safe salvage modality for recurrent, radio-resistant ONB, allowing for high tumor doses while sparing critical brain structures. The discordant response between the tumor rim and necrotic core highlights the challenge of heterogeneity, supporting the rationale for fractionated BNCT protocols and careful surveillance of regional nodal basins.

## Introduction

Olfactory neuroblastoma (ONB), or esthesioneuroblastoma, is a rare malignant neoplasm of the sinonasal tract arising from the olfactory neuroepithelium [1]. While it accounts for only three to 6% of sinonasal malignancies, ONB is characterized by an aggressive biological behavior with a propensity for local invasion into the orbit, cribriform plate, and intracranial vault [2]. Due to nonspecific early symptoms such as epistaxis and nasal obstruction, patients frequently present with advanced-stage disease (Kadish stage C or D).

The management of ONB typically involves surgical resection followed by radiotherapy; however, recurrence rates remain high despite multimodal therapy [3, 4]. Treating recurrent ONB is particularly challenging, especially when the tumor involves complex skull base structures previously subjected to high-dose irradiation. In this setting, the proximity of critical organs severely limits the utility of conventional photon radiotherapy. Consequently, in cases of re-irradiation involving infiltrative tumors near critical brain structures, even particle therapy carries a risk of cumulative toxicity and radiation necrosis [5].

Boron neutron capture therapy (BNCT) offers a distinct radiobiological advantage for such refractory cases. BNCT relies on a biochemical targeting mechanism wherein boron-10 compounds are selectively uptaken by tumor cells [6]. Upon irradiation with thermal neutrons, the boron capture reaction releases high-linear energy transfer alpha particles and lithium-7 nuclei. These particles have a path length of approximately 5 to 9 μm, roughly the diameter of a single cell, allowing for the selective destruction of tumor tissue while sparing adjacent normal cells [6].

This report describes the successful management of a patient with multiply recurrent, intracranial ONB who had exhausted all conventional surgical and radiotherapeutic options. We detail the clinical outcomes of a two-course BNCT protocol and discuss how intratumoral heterogeneity influences boron uptake and treatment efficacy, providing a rationale for fractionated approaches in large, necrotic tumors.

## Case presentation

**Figure 1:**
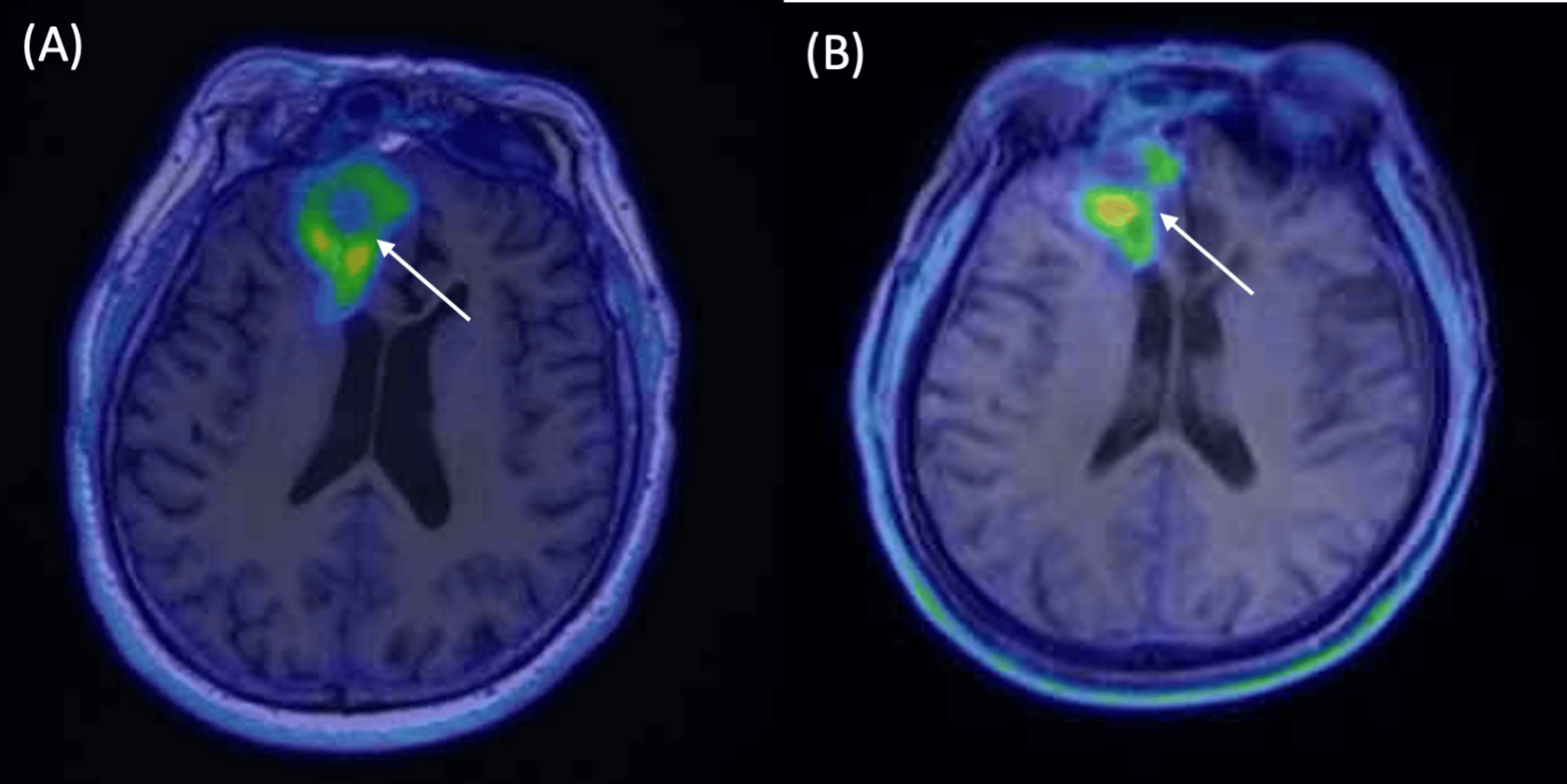
18F-fluciclovine PET evaluation. (A) Pre-1st BNCT Baseline (January 4, 2024); (B) Post-1st BNCT Follow-up (November 14, 2024). The white arrowheads indicate the regions of high metabolic tumor activity, which surround a central area of necrosis. BNCT: boron neutron capture therapy.

A 50-year-old male with a history of recurrent ONB presented to our department for salvage therapy evaluation. Originally diagnosed and surgically resected in 1992, he experienced a significant recurrence in 2013, which was managed with surgical resection followed by intensity-modulated radiation therapy (IMRT). In February 2021, the patient suffered a recurrence involving intracranial invasion. Despite undergoing multiple salvage procedures, including endoscopic endonasal approaches and craniotomies between March 2021 and November 2023, complete tumor control could not be achieved. Follow-up Fluorodeoxyglucose-Positron Emission Tomography (FDG PET) imaging in January 2024 identified persistent recurrence with evident central necrosis (Figure 1A; compare with post-treatment Figure 1B).

**Figure 2:**
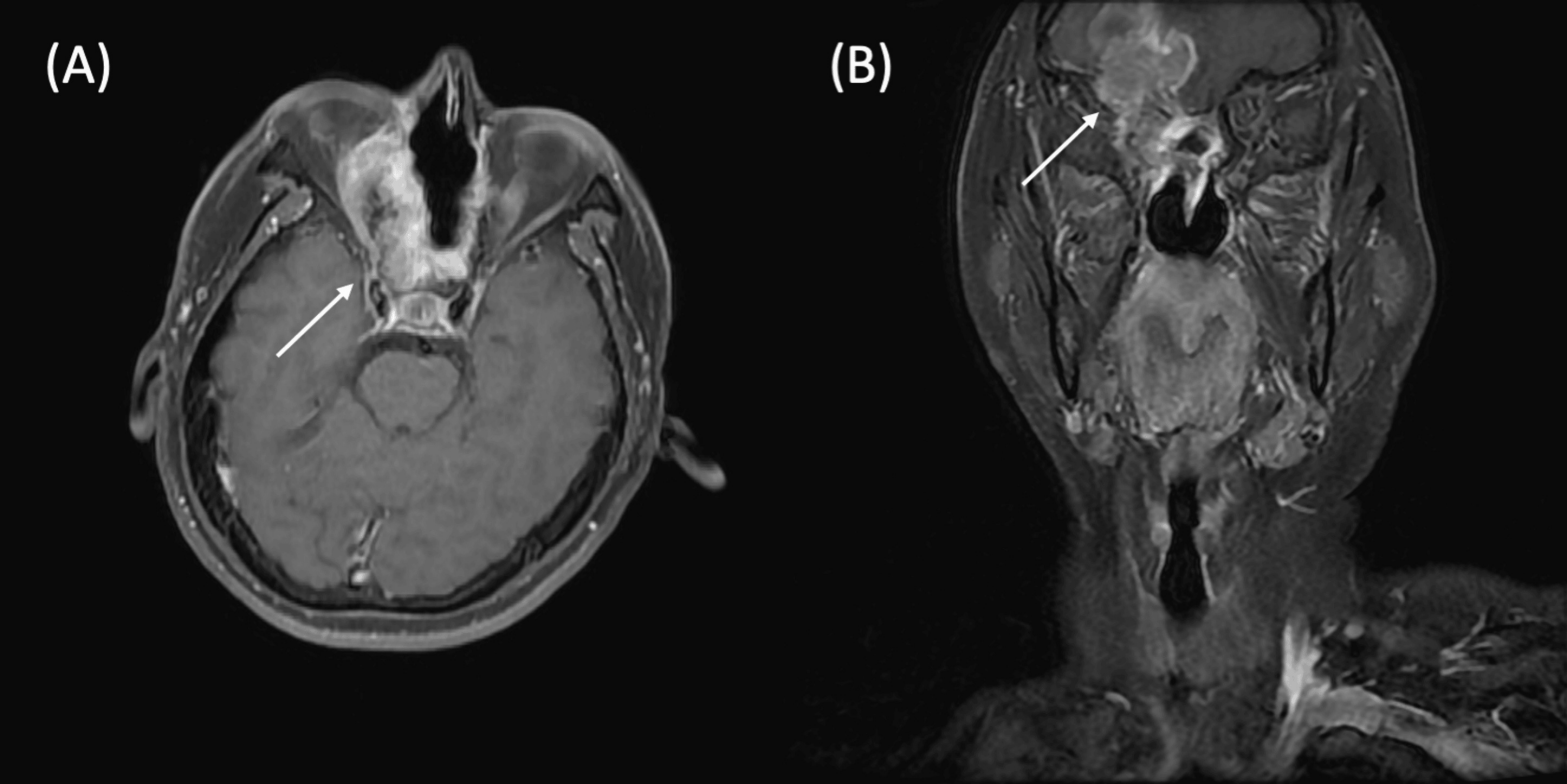
Axial and coronal view of contrast-enhanced T1 MRI at pre-treatment baseline. (A) Axial view and (B) Coronal view showing the pre-treatment baseline. White arrows indicate the location of the recurrent tumor mass.

By March 2024, MRI revealed a massive recurrent tumor measuring 7.25 × 4.11 cm, extending through the cribriform plate into the right frontal lobe (Figure 2). Given the exhaustion of conventional surgical and photon radiotherapy options, the patient was enrolled for BNCT. 18F-fluciclovine PET imaging was utilized to determine the tumor-to-normal (T/N) ratio for tumor delineation and dose calculation. This approach is supported by recent clinical investigations demonstrating the feasibility of using 18F-fluciclovine as a surrogate for assessing boron uptake in recurrent intracranial malignancies [7]. The preparation of BPA and the neutron source were consistent with those described in previous studies [8]. The compound biological effectiveness (CBE) factors adopted were 3.8 for tumors [9], 4.9 for mucosa [10], 1.35 for nerves [11], and 2.27 for bones [12]. Treatment planning was performed using the Simulation Environment for Radiotherapy Applications (SERA). SERA reconstructs patient geometry from CT images to accurately calculate the complex mixed-field dose components, including boron, thermal neutron, fast neutron, and gamma ray doses.

**Figure 3:**
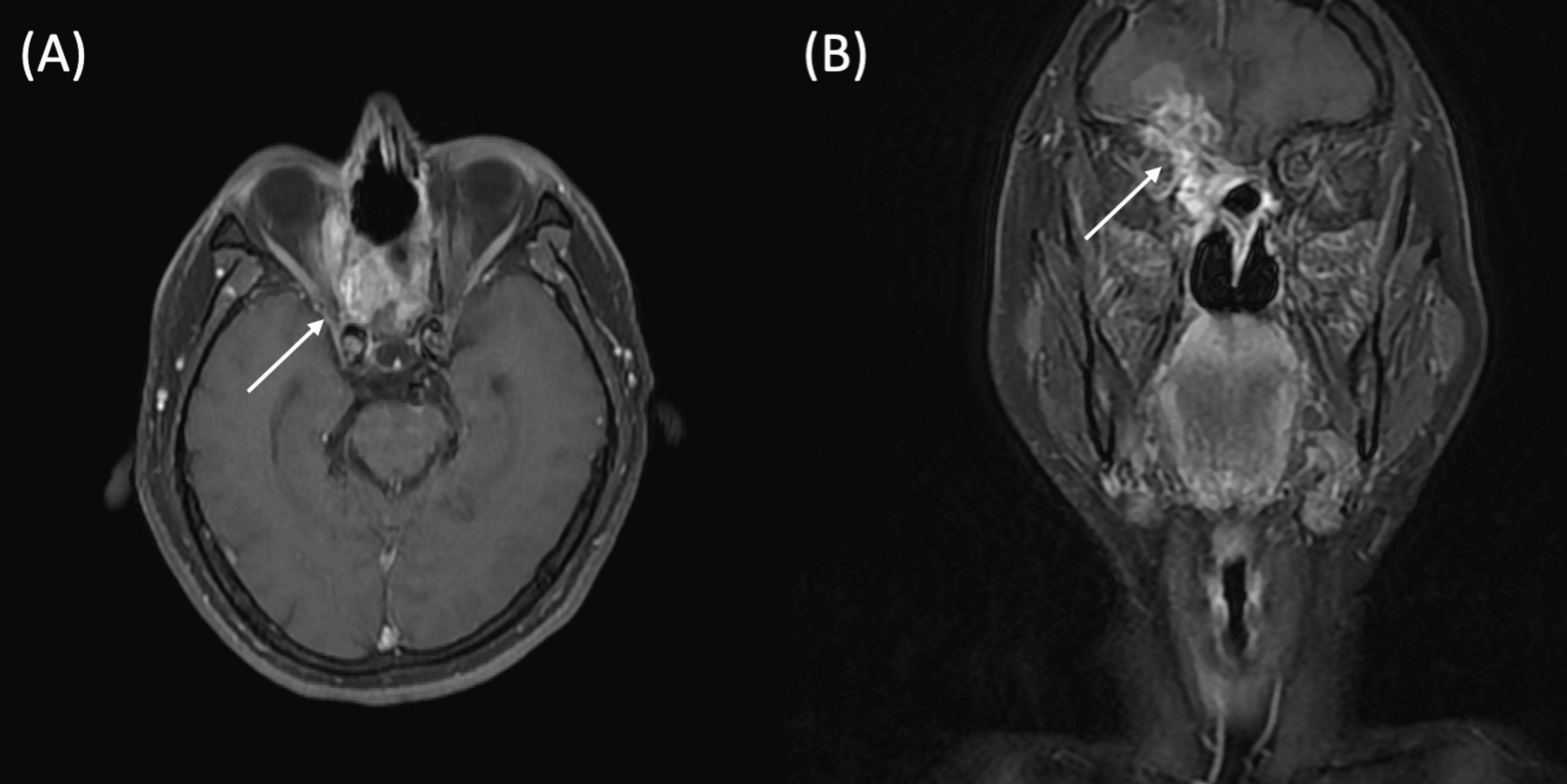
Axial and coronal view of contrast-enhanced T1 MRI eight months post-1st BNCT. (A) Axial view and (B) Coronal view obtained eight months after the first BNCT course. White arrows indicate the location of the recurrent tumor mass and post-BNCT cystic change. BNCT: boron neutron capture therapy.

The first course of BNCT was administered on April 12, 2024, achieving a tumor maximum dose (Dmax) of 49.2 Gy-Eq. The treatment was well-tolerated with only grade I mucositis. A follow-up MRI in December 2024 demonstrated significant tumor regression to 2.40 × 2.02 cm (Figure 3). Crucially, repeated biopsies of the right nasal wall and orbital wall yielded only granulation tissue. Nevertheless, 18F-fluciclovine PET imaging continued to indicate residual metabolic tumor activity (Figure 1B). Encouraged by the excellent regression achieved with the initial treatment, the patient opted for re-irradiation, and a second course of BNCT was administered on March 19, 2025, delivering maximum and mean tumor doses of 40.73 Gy-Eq and 36.91 Gy-Eq, respectively. The decision to proceed with a second BNCT course was reached at a multidisciplinary team (MDT) conference. The panel concluded that re-irradiation was feasible given the exhaustion of surgical and photon options, and the favorable safety profile observed in the first course, where doses to organs-at-risk remained well below tolerance (normal brain avg: 2.46 Gy-Eq). Thus, a second course was deemed to offer the best therapeutic ratio with manageable cumulative toxicity risks.

**Figure 4:**
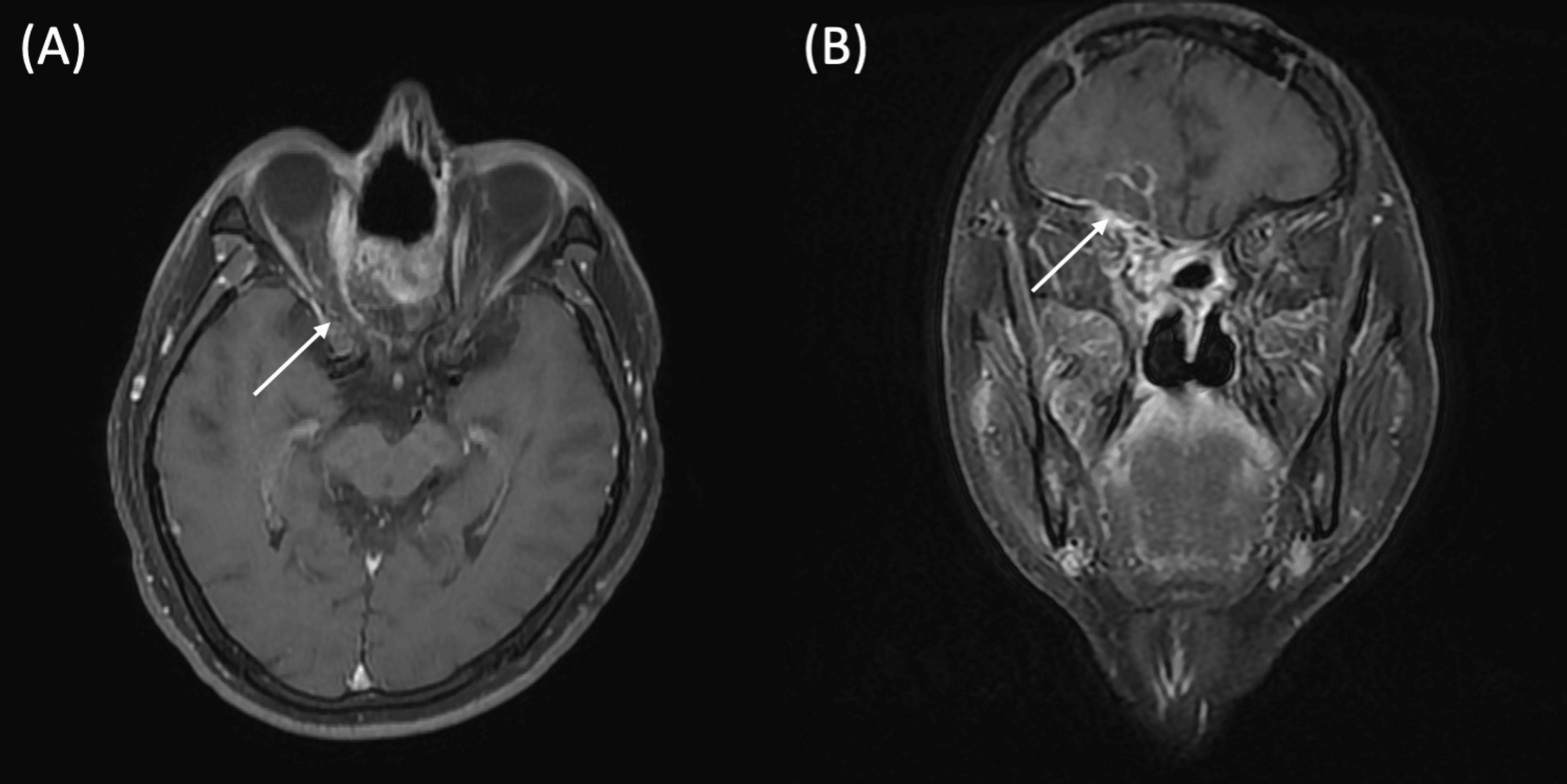
Axial and coronal view of contrast-enhanced T1 MRI six months post-2nd BNCT. (A) Axial view and (B) Coronal view obtained six months after the second BNCT course. White arrows indicate the location of the post-BNCT cystic change. BNCT: boron neutron capture therapy.

Surveillance MRI obtained in September 2025, six months post-reirradiation, revealed a near-complete response at the primary surgical bed. The previously identified solid tumor mass had resolved, leaving only cystic cavities and necrotic changes in the treatment field (Figure 4). However, the scan revealed a new 2.3 cm enlarged cervical lymph node at the left level II, indicating that despite excellent local control, BNCT showed limited efficacy in suppressing out-of-field regional metastases. Given the imaging characteristics consistent with progression, this lesion was diagnosed clinically as a regional metastasis. Histopathological confirmation was deferred due to the patient's preference for a period of close observation. Multidisciplinary evaluation has outlined potential salvage modalities, including neck dissection or photon radiotherapy, which remain under consideration pending further disease evolution. Ophthalmic evaluation remained stable relative to his post-2013 baseline, showing optic disc pallor consistent with chronic treatment-related sequelae. As of November 2025, the patient remains clinically stable with sustained local control of the radio-resistant skull base tumor despite regional nodal progression.

## Discussion

The management of recurrent olfactory neuroblastoma presents a formidable challenge, particularly in patients with intracranial invasion who have exhausted standard treatment modalities. While the combination of surgery and radiotherapy remains the cornerstone of first-line therapy, recurrence rates for Kadish C and D stages are significant [2]. In the setting of re-irradiation, the proximity of critical structures in the brain often limits the deliverable dose of conventional photon therapy. Although particle therapies like proton beam therapy and carbon ion radiotherapy offer superior dose distribution due to the Bragg peak effect, they still carry a risk of cumulative toxicity, including radiation necrosis [13, 14].

BNCT offers a unique biological advantage in this salvage setting. Unlike proton or carbon ion therapy, which rely on physical depth-dose distributions characterized by the Bragg peak [15], BNCT exploits a biochemical targeting mechanism [6]. This distinction is critical in the salvage setting. In this case, the dosimetric data highlight the safety profile of BNCT for intracranial recurrences (Table 1). Despite the patient undergoing two separate courses of BNCT, the average doses delivered to the normal brain were remarkably low (2.46 Gy-Eq in the first course and 3.69 Gy-Eq in the second). This "brain-sparing" capability allowed for the safe delivery of a high cumulative tumor dose (Dmax 49.2 Gy-Eq and 40.73 Gy-Eq, respectively) without inducing neurological deficits or high-grade toxicities, a feat that would be difficult to achieve with conventional re-irradiation.

**Table 1:** BNCT radiation dose distribution (Gy-Eq). BNCT radiation dose distribution (Gy-Eq). Vol = Volume (ml); Dmax = Maximum Dose; Dave = Average Dose; Dmin = Minimum Dose; 1st = First BNCT course; 2nd = Second BNCT course; "CN2 = Optic nerve". CBE factors used: Tumor = 3.8; Mucosa = 4.9; Nerve = 1.35; Bone = 2.27. BNCT: boron neutron capture therapy

Organ	Vol (1st)	Dmax (1st)	Dave (1st)	Dmin (1st)	Vol (2nd)	Dmax (2nd)	Dave (2nd)	Dmin (2nd)
Tumor	49.44	49.2	36.78	23.8	5.49	40.73	36.91	28.47
Mucosa	131.44	16.03	10.32	4.58	146.27	17.98	8.22	3.57
Skull	1264.83	8.15	2.09	0.66	1591.14	10.13	1.87	0
Brain	1199.03	7.63	2.46	0.32	1230.24	10.27	3.69	0.63
Rt Eye	13.13	7.99	6.38	4.17	11.48	6.91	4.98	2.85
Lt Eye	11.64	7.74	6.55	4.54	10.99	3.41	2.65	1.78
Rt CN2	3.4	7.74	5.6	2.85	2.19	6.9	5.1	3.71
Lt CN2	2.89	7.44	5.36	2.94	2.47	3.83	3.53	3.15

A critical teaching point from this case is the impact of intratumoral heterogeneity on BNCT efficacy. While the pre-treatment 18F-fluciclovine PET scan indicated a favorable T/N ratio of 3.68, this value represented the peak metabolic activity. As observed in Figure 2, the central region of the massive tumor showed hypointense signals consistent with necrosis, which likely resulted in suboptimal boron uptake in the tumor core. This heterogeneity explains why, despite significant shrinkage, residual activity persisted after the first single-fraction treatment. The use of a second BNCT course proved effective in targeting these radio-resistant remnants. This observation strongly supports the rationale for fractionated BNCT protocols for large, heterogeneous tumors. Fractionation may allow for the re-oxygenation of hypoxic regions and the redistribution of the boron compound into previously poorly perfused areas, thereby overcoming the limitations of single-fraction treatment.

While BNCT achieved excellent local control of the primary skull base tumor, converting a solid mass into cystic cavities, the patient subsequently developed a new cervical lymph node metastasis. Recent preclinical and translational data have suggested that BNCT may induce antitumor immune responses, potentially exerting effects beyond the local treatment field via immune-modulatory mechanisms [16]. However, in this case, the development of regional metastasis suggests that such abscopal effects were insufficient to control subclinical disease in the non-irradiated neck. This pattern of failure suggests that while BNCT is highly effective locally, it cannot address subclinical regional micrometastases that fall outside the neutron irradiation field. Literature suggests that prophylactic neck irradiation can improve local control in ONB [17]. In the context of salvage BNCT for advanced-stage disease, clinicians should carefully consider whether to combine local BNCT with elective neck irradiation or systemic therapies to address potential microscopic disease, even if the neck appears clinically negative at the time of recurrence. Finally, this report is limited by its nature as a single case study and the relatively short follow-up period.

## Conclusions

This study provides proof-of-concept that BNCT can serve as a potent and safe salvage modality for multiply recurrent, intracranial ONB. The heterogeneous response observed here underscores the necessity of detailed functional imaging in treatment planning and advocates for further research into fractionated BNCT regimens to maximize therapeutic gain.
